# Investigation of Blade Printing Technique for Nano-Structuring Piezoelectric Polymer Ink in a Porous Anodic Aluminum Oxide

**DOI:** 10.3390/polym17212839

**Published:** 2025-10-24

**Authors:** Tsvetozar Tsanev, Mariya Aleksandrova

**Affiliations:** Department of Microelectronics, Technical University of Sofia, 1756 Sofia, Bulgaria; m_aleksandrova@tu-sofia.bg

**Keywords:** energy harvesting, piezoelectric polymer, nanostructuring, anodic aluminum oxide, nanorods, blade printing

## Abstract

In this work, we investigated the use of a piezoelectric flexible device for energy harvesting. The main goal of the study was to fill the nanostructured pores of anodic aluminum oxide (AAO) films with piezoelectric polymer (PVDF-TrFE) via a modified conventional screen printing technique using blade printing. In this way, it is possible to obtain a composite from nanostructured thin films of polymer nanorods that shows improved charge generation ability compared to other non-nanostructured composites or pure (non-composite) aluminum with similar dimensions. This behavior is due to the effect of the highly developed surface of the material used to fill in the AAO nanopore template and its ability to withstand the application of higher mechanical loads to the structured piezoelectric material during deformation. The contact blade print filling technique can produce nanostructured piezoelectric polymer films with precise geometric parameters in terms of thickness and nanorod diameters, at around 200 nm, and a length of 12 μm. At a low frequency of 17 Hz, the highest root-mean-square (RMS) voltage generated using the nanostructured AAO/PVDF-TrFE sample with aluminum electrodes was around 395 mV. At high frequencies above 1700 Hz, the highest RMS voltage generated using the nanostructured AAO/PVDF-TrFE sample with gold electrodes was around 680 mV. The RMS voltage generated using a uniform (non-nanostructured) layer of PVDF-TrFE was 15% lower across the whole frequency range.

## 1. Introduction

Nowadays, autonomous power sources are needed to run various types of low-power electronic equipment (sensors, actuators, wearable electronics, etc. [1]). These devices often operate in environments where traditional power supplies are impractical or undesirable, necessitating the use of self-sufficient energy harvesting methods. The development of high-performance piezoelectric devices thus depends on improvements to their functional characteristics, such as their output voltage, energy conversion efficiency, and mechanical durability. To achieve this, various optimization strategies are being explored. One key avenue involves the utilization of environmentally friendly and biocompatible materials that do not emit toxic substances, aligning with the goals of sustainable and safe device deployment, particularly in biomedical applications, but have similar physical and functional characteristics to conventional (lead-based) materials [2]. Piezoelectric devices often need to be implanted into human tissues, which requires biocompatibility between the materials of the device and the human body [3]. For instance, replacing conventional lead-based piezoelectric materials with lead-free alternatives, such as potassium sodium niobate or bismuth-based compounds, is critical. These materials are designed to match the piezoelectric coefficients (d_33_, d_13_) of their lead-containing counterparts, ensuring comparable electromechanical performance while mitigating health and environmental concerns [4]. Another avenue involves the use of flexible substrates such as polyethylene naphthalate (PEN) or polyethylene terephthalate (PET), which confer mechanical flexibility and conformability to devices. These substrates are often combined with various structuring approaches, such as nanostructuring, to enhance the electric response or mechanical robustness of piezoelectric layers [5]. Nanostructuring techniques—such as the incorporation of nanowires, nanopillars, or nanosized pores—serve to increase the active surface area, enhance polarization, and facilitate charge separation, thereby substantially improving the device’s electromechanical coupling efficiency. Furthermore, the electrode configuration and material selection—ranging from conductive polymers to metal nanowires—are also optimized to maximize charge collection and overall device performance. Different electrode topologies influence the distribution of electric fields within the piezoelectric layer, impacting parameters such as the charge density and output voltage [6]. Recent technological advances include the nanostructuring of piezoelectric materials to further boost device performance using techniques such as anodic aluminum oxide (AAO) templating [7]. This facilitates the fabrication of ordered nanoporous structures that can host piezoelectric inks or composites [8].

A versatile, fully operational piezoelectric generator has been demonstrated by Kim et al. [9], who grew ZnO nanorods on woven polyester substrates coated with gold. The assembly was completed with the compression of an additional gold-coated polyester layer on top, serving as the upper electrode. This configuration enabled electrical contact at both the base and the tip of the nanorods, allowing the piezoelectric polarization—generated by the strain during cloth bending—to effectively produce a voltage in an external circuit. An enhancement in output was accomplished by inserting a 40 µm polyethylene spacer between the nanorods and the top electrode, which yielded an open-circuit voltage of 4 V and a short-circuit current density of 0.15 μA/cm^2^ when subjected to acoustic vibrations.

Lee et al. [10] explored the potential of a flexible piezoelectric generator utilizing hexagonal boron nitride (h-BN) nanoflakes. These nanoflakes (lateral dimension, 0.82 µm; thickness, 25 nm) were produced via mechanochemical exfoliation and transferred onto a plastic substrate patterned with electrical electrodes. The mechanical deformation of a single h-BN nanoflake generated alternating electrical outputs, with a voltage of 50 mV and a current of 30 pA. Additionally, a flexible piezoelectric device based on h-BN nanoflakes was constructed, providing a voltage of 9 V, a current of 200 nA, and an output power of 0.3 μW.

Incorporating porosity into piezoelectric materials represents another effective approach to enhancing their performance. Roscow et al. [11] produced porous barium titanate (BT) using polyethylene glycol as a volatile pore-former. The structure evolved from a 3–0 configuration (consisting of isolated pores) to a 3–3 structure (features interconnected pores) as the porosity increased. The highest energy harvesting figure of merit recorded was 2.85 pm^2^/N at 60% porosity, which is three times greater than that of non-porous samples. This improvement was attributed to a decrease in permittivity at this porosity level, with only a modest reduction in the piezoelectric coefficient d_33_.

Compared to their inorganic counterparts, piezoelectric polymers are inherently flexible, robust, and easier to process, making them advantageous in a wide range of applications [12]. A copolymer of PVDF with trifluoroethylene, known as P(VDF-TrFE), is frequently used as a coating for energy harvesting elements [13]. Although the piezoelectric effects in polymers are generally weaker than those observed in ferroelectric ceramics, polymers benefit from low permittivity and minimal acoustic impedance. They are produced as large-area sheets (or thin films) and are flexible, lightweight, and cost-effective. Their high elastic compliance allows them to conform to curved surfaces, enhancing their versatility. Electrospinning, template wetting, and nanoimprinting lithography are examples of techniques for nanopatterning organic piezoelectric materials [14]. Each method involves trade-offs between pattern control, material compatibility, defect risk, and scalability [15]. Various technologies for piezoelectric nanostructuring materials have been reported. Usually, they are relatively complex with many technological steps, for example, lithography and pulsed laser deposition, sputtering deposition, and chemical bath deposition [16,17,18], facing limitations such as vacuum requirements, complex parameter control, challenges with scalability and large-area deposition, or waste products. Blade printing can potentially overcome some of these challenges, offering a simpler, less expensive, and more scalable method for large-area coatings.

These nanostructured layers exhibit enhanced surface roughness, increased polarization sites, and improved mechanical–electrical coupling, leading to higher energy conversion efficiencies. Specifically, integrating piezoelectric inks into nanostructured anodic aluminum oxide (AAO) templates allows for precise control over the morphology and nanostructure of the active layers, which is critical in tailoring their functional properties. Using AAO in energy harvesting leads to several compelling advantages, such as a high aspect ratio for the functional elements, a highly ordered, porous nanostructure with tunable pore size, a high surface area, and excellent mechanical stability. A higher aspect ratio for the piezoelectric nanorods generates a higher output voltage. This is related to the length and diameter of nanorods (aspect ratio). Longer nanorods with smaller diameters and good alignment are more easily bent and experience more highly concentrated mechanical stress. For this reason, they generate higher piezoelectric potential [19].

Different aspect ratios between nanorod diameters and lengths have been reported for different piezoelectric materials and deposition technologies. For example, PZT plasma-etched nanostructured nanorods with an aspect ratio of >30:1, produced via an expensive plasma etching process, were reported in [20]. For ZnO nanorods grown on seeded substrates at different molar concentrations, aspect ratios from 3 to 38 have been reported [19]. In the present work, the aspect ratio of PVDF-TrFE nanorods grown in a porous aluminum oxide matrix was >60:1, which can be considered high compared to results reported in the literature. This explains how they achieve competitive nanogenerator behavior despite the lower inherent piezoelectric coefficient of the piezoelectric polymeric ink compared to inorganic piezoelectric materials.

These properties make the AAO an ideal template for fabricating nanostructured piezoelectric materials with precise control over morphology and size. The highly ordered pore channels of AAO enable the growth of nanorods, nanotubes, or nanowires with uniform dimensions, leading to enhanced piezoelectric properties due to an increased surface-to-volume ratio and optimized stress distribution. A typical nanogenerator comprising ≈1010 highly crystalline, self-poled, aligned nanowires spanning ≈2 cm^2^ was shown to produce a peak output voltage of 3 V at 5.5 nA in response to low-level vibrations [21].

In this work, we aimed to develop a conventional yet effective manufacturing process, specifically, blade printing, to produce high-performance nanostructured composite layers based on AAO templates and piezoelectric ink (PEI). By employing this technique, we aimed to demonstrate the feasibility of fabricating localized nanostructures in a scalable manner and to evaluate their electrical response and functional integrity. This approach aligns with the goal of developing piezoelectric energy harvesting devices that are suitable for applications requiring flexible, biocompatible, and environmentally safe materials, contributing to the broader field of autonomous power generation systems.

## 2. Materials and Methods

In this paper, the conventional technology of screen printing is adapted and a blade printing technique is proposed for applying piezoelectric ink (PEI) to the nanopores of a thin film layer of anodic aluminum oxide (AAO) to create a composite nanostructured piezoelectric generator element with superior electromechanical performance.

### 2.1. AAO Deposition Method

**Figure 1:**
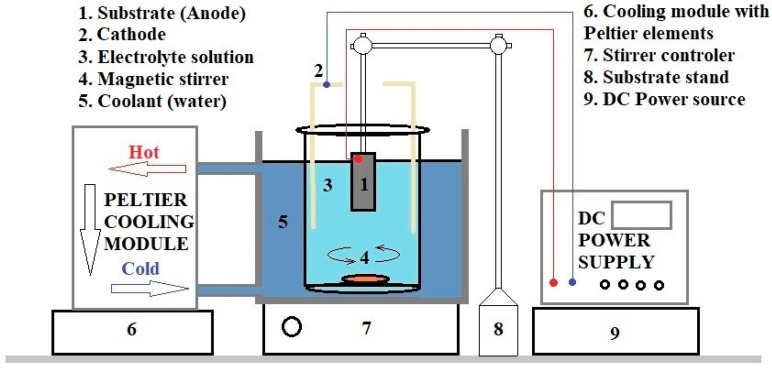
Schematic presentation of AAO deposition system.

To create nanoporous thin-film membranes, AAO was grown using an aluminum substrate with a purity of 99.3% in an electrolyte solution with 5% orthophosphoric acid and a process temperature of 8 °C (Figure 1). Layers of different thicknesses, 4, 6, 8, 12, and 16 μm, were anodized for different periods of time, with nanopore diameters between 180 and 200 nm. The applied voltage was 135 V, and the current ranged from 15 to 35 mA for a substrate with an anodized surface area of 5.25 cm^2^. Under these conditions, the relation between the anodization time and growth rate of AAO templates was determined to be 0.1 μm /min. The AAO sample growth conditions are summarized in Table 1.

### 2.2. Blade Printing of PEI in AAO

**Figure 2:**
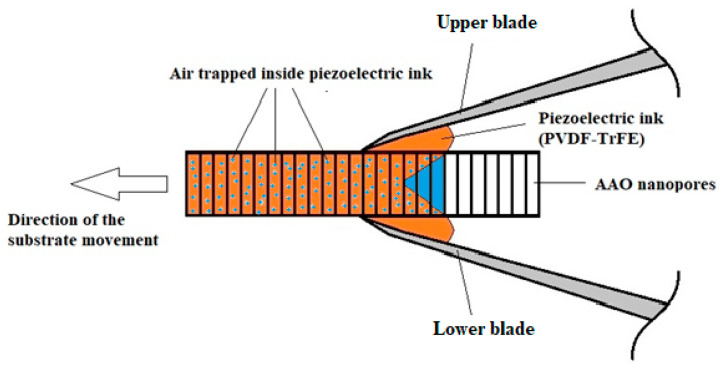
Schematic presentation of the blade printing process with parallel blades and air trapped in the nanopores bulk (unacceptable).

**Figure 3:**
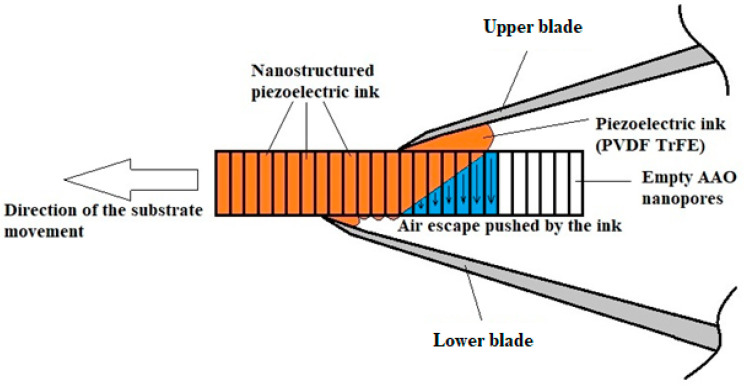
Schematic presentation of the blade printing process with unparallel air escape from nanopores bulk during the filling process (acceptable).

Screen printing methods are widely used in the manufacturing of microelectronic devices, as well as other applications. These methods are cheap and reliable once the correct parameters have been defined. However, they are not directly applicable to the proposed harvester; therefore, we suggest implementing suitable adaptations via blade printing (Figure 2 and Figure 3).

The piezoelectric polymer that is used in this research is a commercial product, Nanopaint’s PEInk01NP (Vale de São Cosme, Portugal) [22]. Its basic parameters, according to the technical specification, are provided in Table 2 and Table 3. This ink can be applied via spin-coating to a wide range of substrates.

### 2.3. Preparation and Characterization of Energy-Harvesting Element

**Figure 4:**
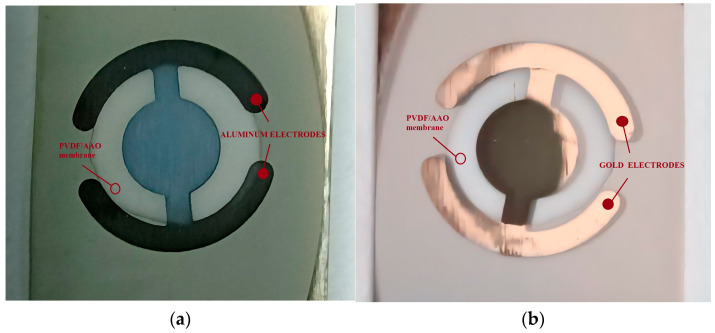
PVDF-TrFE/AAO nanostructured membranes (**a**) with aluminum electrodes, (**b**) with gold electrodes.

To achieve top Ohmic contacts, two types of electrodes—Al and Au—were grown as thin films through a two-sided shadow mask via vacuum thermal evaporation and DC sputtering, respectively. Metals with a purity of 99.998% were used. To avoid the thermal deflection of the AAO membranes and the induction of thermal stress that may affect the durability of the template, the deposition conditions were as follows: for the evaporation, a base pressure of 1 × 10^−5^ Torr and a current of 60 A flowing through the crucible for 15 s, and for the sputtering, an argon pressure 2.5 × 10^−2^ and a sputtering current of 30 mA for 60 s. Photographs of the samples produced with aluminum and gold electrodes are shown in Figure 4.

The samples underwent scanning electron microscopy (SEM) analysis to evaluate coating uniformity, coverage consistency, and defect formation, as well as how these properties varied with different deposition parameters. Energy-dispersive X-ray analysis (EDX) was applied to check the chemical composition of the filler or the distribution of the piezoelectric ink along the thickness of the AAO (i.e., the length of the PVDF-TrFE nanorods). SEM and EDX measurements were conducted using Lira/Tescan and Bruker AXSMicroanalysis GmbH, Ettlingen, Germany, respectively.

The piezomodulus d_33_ of the AAO samples filled with PVDF-TrFE was directly measured using the Quasi Static Piezoelectric Constant d_33_ Meter PKD3-4000, Polyk, North Philipsburg, Pennsylvania, USA. The piezometer allows the loaded mechanical force to be varied in incremental steps of 1 N, while the frequency of vibration at the end of the tip probe is fixed at 110 Hz. The goal in performing this measurement was to compare the values of the d_33_ modulus with the ink manufacturer’s data. In this way, it can be proven that the piezoelectric properties of the polymeric ink are not compromised during the blade printing process. Moreover, it provides an indirect proof of concept regarding the full penetration of the ink into the nanopores and reliable contact with the top and the bottom electrodes from the front end and the back end of the samples.

Five PVDF-TrFE/AAO membrane samples were evaluated, and their measurements were statistically averaged to enhance the reliability and validity of the results.

A series of cyclic bending stimuli, involving both tension and compression, were performed using a custom-designed, beam-shaped testing apparatus. A signal generator with a controlled frequency range from 17 Hz to 200 kHz and a voltage from 0 V to 15 V was used to apply a signal to an electromagnetic coil with an attached cantilever beam. Voltage variations led to different mechanical forces acting on the structures. When tested, each sample was attached to the beam to transmit mechanical oscillations from the coil. The piezoelectric voltage was measured for specific forces (from 10 g to 110g) and frequencies (from 17 Hz to 17 kHz) applied to all the piezo structures in order to compare the signal generated by each sample. A detailed description of the setup and the corresponding standard for the application of the bending stimuli is given elsewhere [24].

## 3. Results and Discussion

**Figure 5:**
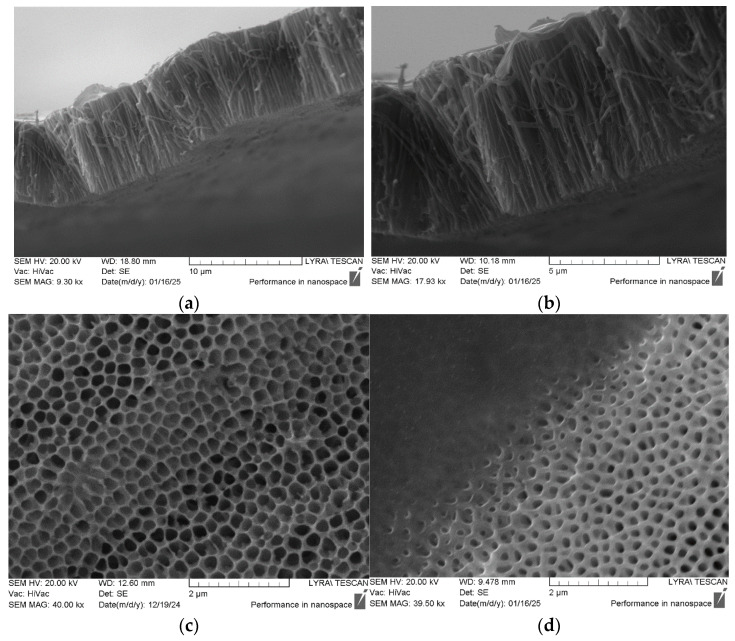
(**a**) SEM images and (**b**) cross-section view of AAO thin film and PVDF-TrFE nanorods formed in the oxide nanopores with different magnifications; (**c**) top view of partially filled pores; (**d**) top view with two separate zones, fully filled pores with PEI and fully unfilled pores.

Figure 5 presents SEM images of the AAO templates with the piezoelectric material (PVDF-TrFE) incorporated. The images allow the visualization of the aspect ratio (length to diameter) of the nanorods and confirm their successful integration into the template. The aspect ratio obtained for polymer nanorods was >60:1, which can be considered relatively high compared to reported values [20]. The SEM images confirm that the blade printing technique effectively introduces the piezoelectric material into the AAO pores.

The SEM images in Figure 5 show that piezoelectric nanorods are formed from PVDF-TrFE piezoelectric ink and nanostructured in the pores of the AAO with a diameter around 200 nm. This is achieved through the use of a PEI blade printing technique on the porous aluminum oxide layer. This method demonstrated the full penetration of the piezopolymeric ink into the volume of the oxide layer and the absence of excessive material on the top of the oxide templating structure. This was achieved thanks to the contact method of deposition between the blade and the oxide layer. Thus, full penetration without excessive loss (non-nanostructured material) from both sides of the porous structure was achieved. In this way, only the nanostructured piezo material is electrically characterized. This represents an advantage, because, very often, thin-film devices cannot be fully nanostructured in terms of their internal geometry and volume.

Our results clearly indicate that the blade printing technique enables the PVDF-TrFE ink to penetrate well into the AAO nanopores (Table 4). This is crucial in creating a functional composite material where the piezoelectric properties of the polymer can be effectively utilized. The reasonably uniform diameter and distribution of the nanorods are indicative of a well-controlled templating process. This uniformity contributes to predictable and enhanced piezoelectric performance.

**Figure 6:**
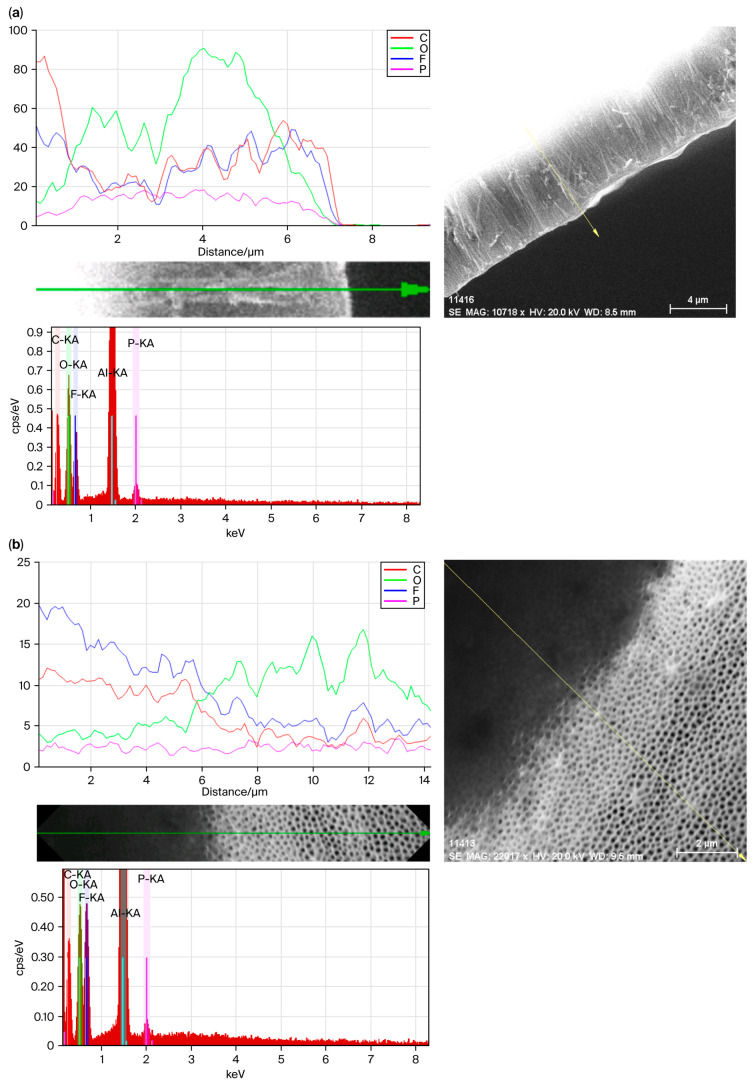
EDX of AAO filled with PVDF-TrFE via blade printing. (**a**) Cross-sectional view of filled nanopores; (**b**) top plane view of the same sample with two zones comprising fully filled and empty nanopores.

The EDX results are presented in Figure 6 for the cross-section and the top of the composite samples. The primary element is the AAO template. We expected a strong, relatively uniform Al signal across the cross-section and top surface, a key element in PVDF-TrFE. We expected the ‘F’ signal to correlate with the presence and distribution of the polymer nanorods within the AAO pores. The signal would be most intense within the pores. If the blade printing process worked well, then the EDX maps would reveal a strong ‘F’ signal within the pores on SEM images. The ‘Al’ signal might be reduced within pores (compared to the AAO walls), as some of the Al had been displaced by PVDF-TrFE.

The results show that during the nanostructuring of the piezopolymer material, there are no changes in its d_33_ piezomodulus or piezoelectric properties. The measured values are close to the manufacturer’s parameters for the piezomodulus of the PEI (d_33_ = 18–23 pC/N) [18].

**Figure 7:**
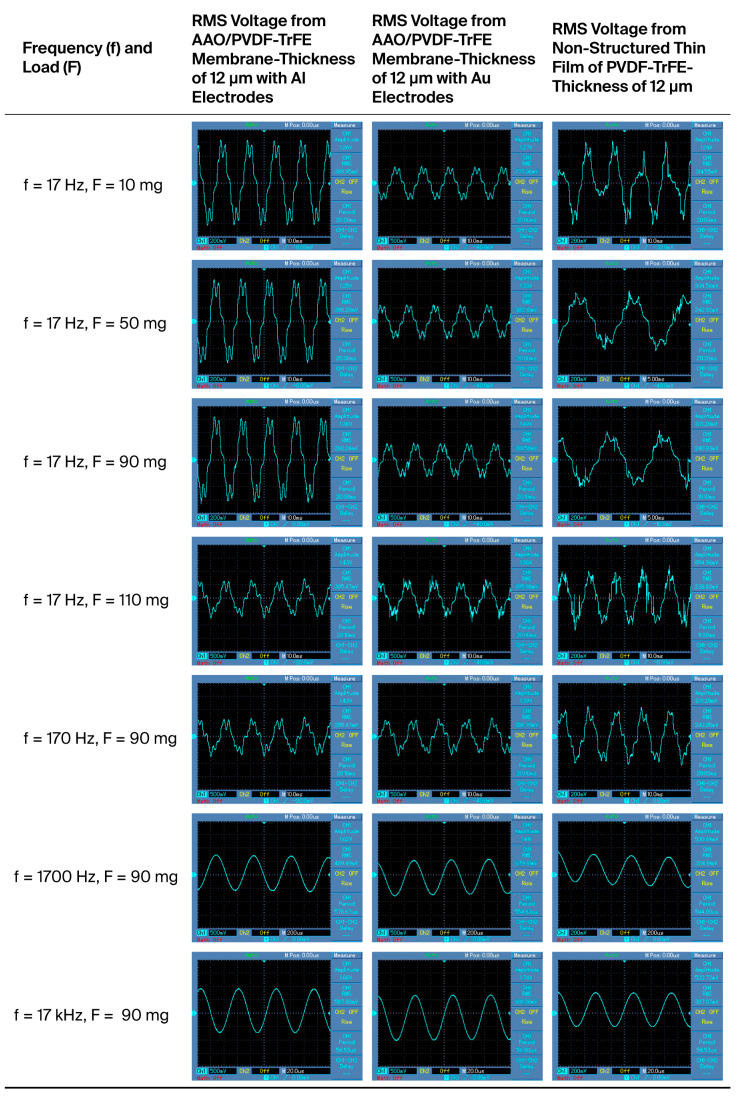
Root mean square (RMS) voltage during oscillation with different mechanical loads and frequencies applied.

Figure 7, Table 5 and Table 6 show the root mean square (RMS) voltage during oscillation under different mechanical loads and frequencies for the samples with two different electrode materials and one pure piezopolymer of the same thickness. Three types of thick piezomembrane were selected with the same thickness as the piezoelectric layer, 12 μm, for oscillation measurements, in order to compare RMS values. Thicker membranes were chosen for a higher output voltage.

**Figure 8:**
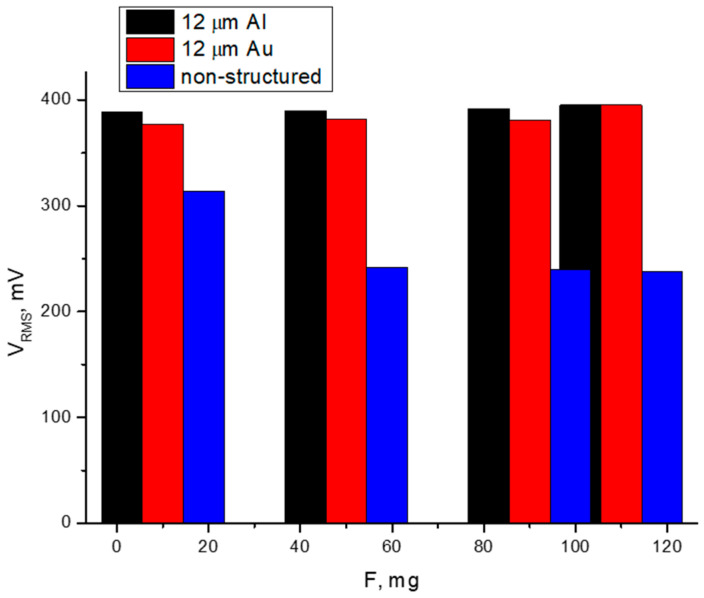
Comparison of the generated voltages from the three devices at a different applied force with the same frequency.

**Figure 9:**
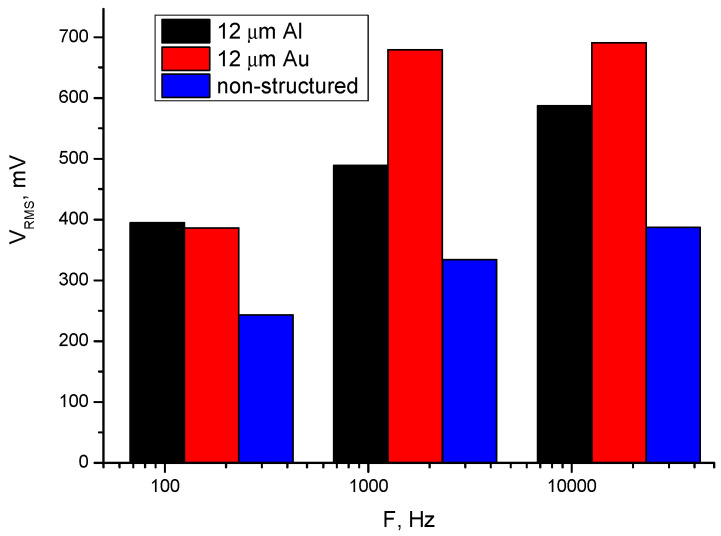
Comparison of the voltages generated using three devices at different frequencies with the same strength of force applied.

It is expected that aluminum-electrode and gold-electrode structures will show a similar piezoelectric response. It is also expected that a non-structured layer made only of piezopolymer will generate lower voltages compared to nanostructured membranes (Figure 8 and Figure 9).

The results show that the samples with aluminum electrodes exhibit better responses at lower frequencies of the various forces applied and lower output voltages at higher frequencies. The nanostructuring of piezoelectric materials enhances the charge carriers’ mobility by more than ten times. This is caused by there being the same order of molecules in the nanorods. Alignment implies fewer defects and therefore fewer traps for charges than bulk materials. A reduction in scattering from defects can lead to an increase in carrier mobility. For this reason, piezoelectric devices with nanostructured rods can operate faster and cover a higher frequency range [25]. Lower contact resistance between the electrodes and the piezocomposite structure also improves the charge carrier’s mobility [26]. Both nanostructured membranes (those with gold and those with aluminum electrodes) exhibit a good piezoelectric response at frequencies above 1700 Hz. The sample with gold electrodes generates a voltage around 20% higher in the same frequency range. The signals of the tested samples show that they are not highly dependable on the mechanical load applied.

The material density affects the stiffness of electrodes on flexible structures. Young’s modulus is related to the lattice density and grain size. For a gold electrode, different values have been reported, depending on the measurement method. In all cases, this value is between 66 and 78 GPa for gold thin films, which is around 10% higher than that of aluminum films [27,28]. This means that the aluminum-electrode structure has a higher elastic compliance and is more capable of generating a higher voltage at the same mechanical load. Variation in the piezoelectric voltage generated may also be related to the material density. Denser electrode material lowers the resonant frequencies, and this might also be the reason for the higher output voltage from a structure with aluminum electrodes operating at a lower applied frequency [29,30]. This may explain the higher output voltages measured for a structure with aluminum electrodes at a lower applied frequency.

The non-nanostructured piezopolymer layer shows less sensitivity in both directions, but, at higher frequencies, the piezoelectric response drops significantly and the difference in the generated voltage is almost double. The higher noise levels in the signal generated from this layer can be ascribed to the absence of AAO matrix in the structure.

## 4. Conclusions

In this research, we assessed the nanostructuring of piezoelectric ink using a modified screen printing technology, namely, blade printing. We demonstrated the successful deposition and nanostructuring of piezopolymer nanorods in a template of anodic aluminum oxide. We also investigated and demonstrated their functionality under loads. The combined SEM and EDX data provide strong evidence for the successful fabrication of nanostructured piezoelectric composites using the blade printing method. The images confirm the formation of well-defined PVDF-TrFE nanorods within the AAO template, which directly correlate with the data for piezoelectric coefficients and values. Piezogenerator structures achieved values around 15% higher for piezoelectric voltage and piezocoefficients compared to the non-structured thin films of the same material. After the nanostructuring of PVDF-TrFE in the pores of the AAO, a similar piezoelectric coefficient d_33_ was achieved to that of the non-structured layer. The piezomodulus does not change after the nanostructuring process or electrode deposition. This confirms that no change occurs in the molecular geometry of the polymer material as a result of the blade deposition process. Possible factors in higher RMS voltages being generated by the nanostructures include the higher mechanical stress experienced by the polymer material and the higher charge carriers and mobility and more highly developed surface in the AAO membrane. The proposed technology is simple and effective and does not require expensive equipment or supplementary tools such as stencils or screens, which are used in screen printing. The deposition of polymer ink is conducted simultaneously from both sides of the substrate (AAO). This halves the manufacturing time. A further advantage is that harmful chemicals are not released during the deposition process of piezoelectric materials, making this technology environmentally friendly.

## Figures and Tables

**Table 1:** Summary of growth conditions for the fabrication of nanoporous AAO.

Applied Voltage DC [V]	Electrolyte Temperature [°C]	Anodization Time [min]	Current [mA]	Layer Thickness [μm]
135	From 7 to 9	20	8	2
135	From 7 to 9	40	15	4
135	From 7 to 9	60	17	6
135	From 7 to 9	80	20	8
135	From 7 to 9	120	30	12

**Table 2:** Polymeric ink properties (manufacturer’s specifications) [23].

Appearance	Clear/transparent
Cure processing	Thermal cure
Solid content (%)	25%
Viscosity	4000–8000 cP

**Table 3:** Piezoelectric/pyroelectric parameters (manufacturer’s specifications) [18].

Piezoelectric coefficient d_33_ (pC/N)	18–23
Pyroelectric coefficient ρ (μC/m^2^.K)	−23
Remnant polarization Pr (mC/m^2^)	80
Curie temperature	135 °C

**Table 4:** Measured d_33_ piezomodulus for samples with different thicknesses from 2 to 12 μm.

Sample Thickness [μm]	d_33_ [pC/N]
2	18.5
4	18.7
6	18.6
8	19.2
12	20.4

**Table 5:** Measured RMS voltage for three samples with variable force (F) and constant frequency (f) of 17 Hz.

F [mg]	12 μm Al Electrode [mV]	12 μm Au Electrode [mV]	12 μm Non-Structured [mV]
10	389	377	314
50	390	382	242
90	392	381	240
110	395	395	238

**Table 6:** Measured RMS voltage for three samples with variable frequency (f) and constant force (F) of 90 mg.

f [Hz]	12 μm Al Electrode [mV]	12 μm Au Electrode [mV]	12 μm Non-Structured [mV]
170	395	386	243
1700	489	679	334
17000	587	691	387

## Data Availability

The original contributions presented in this study are included in the article. Further inquiries can be directed to the corresponding author.
